# Boron Nitride Nanotubes for Spintronics

**DOI:** 10.3390/s140917655

**Published:** 2014-09-22

**Authors:** Kamal B. Dhungana, Ranjit Pati

**Affiliations:** Department of Physics, Michigan Technological University, Houghton, MI 49931, USA; E-Mail: kbdhunga@mtu.edu

**Keywords:** electronic structure, spintronics, spin-valve, ferromagnetic spin ordering, transverse electric field, radial deformation, functionalization, magnetism, tunneling magneto-resistance, spin filtering

## Abstract

With the end of Moore's law in sight, researchers are in search of an alternative approach to manipulate information. Spintronics or spin-based electronics, which uses the spin state of electrons to store, process and communicate information, offers exciting opportunities to sustain the current growth in the information industry. For example, the discovery of the giant magneto resistance (GMR) effect, which provides the foundation behind modern high density data storage devices, is an important success story of spintronics; GMR-based sensors have wide applications, ranging from automotive industry to biology. In recent years, with the tremendous progress in nanotechnology, spintronics has crossed the boundary of conventional, all metallic, solid state multi-layered structures to reach a new frontier, where nanostructures provide a pathway for the spin-carriers. Different materials such as organic and inorganic nanostructures are explored for possible applications in spintronics. In this short review, we focus on the boron nitride nanotube (BNNT), which has recently been explored for possible applications in spintronics. Unlike many organic materials, BNNTs offer higher thermal stability and higher resistance to oxidation. It has been reported that the metal-free fluorinated BNNT exhibits long range ferromagnetic spin ordering, which is stable at a temperature much higher than room temperature. Due to their large band gap, BNNTs are also explored as a tunnel magneto resistance device. In addition, the F-BNNT has recently been predicted as an ideal spin-filter. The purpose of this review is to highlight these recent progresses so that a concerted effort by both experimentalists and theorists can be carried out in the future to realize the true potential of BNNT-based spintronics.

## 1. Introduction

**Figure 1.**
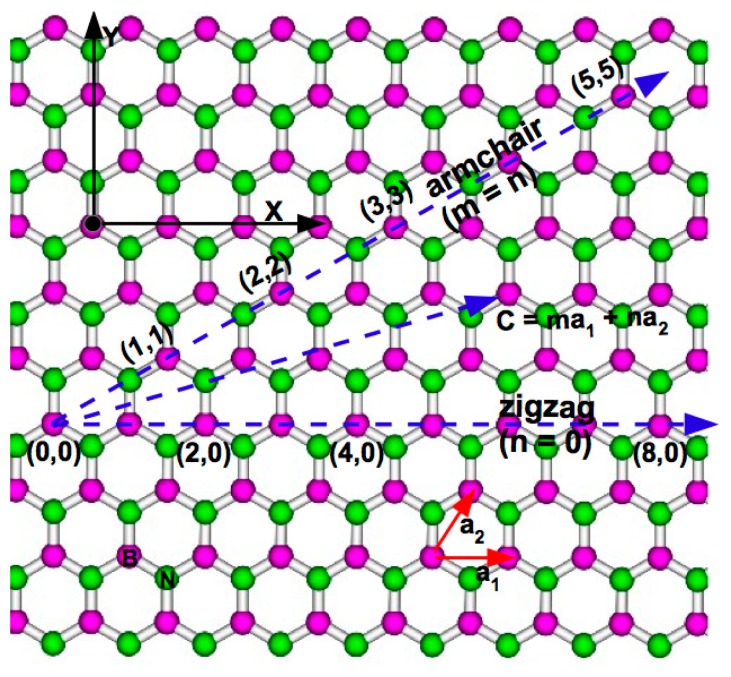
The chiral or the circumferential vector (**C**) in terms of two unit vectors **a_1_** and **a_2_** describes how to roll up the 2D hexagonal BN sheet to form a BN nanotube. The chiral indices (m, n) denote the number of unit vectors along two directions in the honeycomb 2D hexagonal BN lattice. When n = 0, the NT is referred to as the zigzag NT and when n = m, the NT is referred to as the armchair NT.

The boron nitride nanotube (BNNT) has a one dimensional tubular structure. Its existence was first predicted in 1994 [1,2]. Soon after its prediction, it was successfully synthesized by the arc discharge method [3]. The tubular structure of BNNT can be viewed to be formed by rolling a 2D hexagonal BN (h-BN) film analogous to the formation of a carbon nanotube (CNT) from a 2D graphite. Similar to CNTs, the geometry of BNNT is described by a chiral vector, **C** = m**a_1_** + n**a_2_**, denoted by (m, n), which connects the two crystallographic equivalent sites on the h-BN sheet (Figure 1). BNNTs can have single-walled or multi-walled structures, as observed for CNTs, but in a multi-walled BNNT structure, the chirality of the inner shell matches that of the outer shell [4]. The B-N bonds in BNNT are formed by *sp^2^* hybridization, like the C-C bonds in CNTs [5]. Unlike the electronic properties of CNTs, which depend upon the chiral indices (m, n), the electronic properties of BNNTs are found to be independent of chirality [5]. This structurally insensitive electronic property of BNNTs is advantageous for their practical applications, because separating these tubular structures based on their chirality is prohibitively difficult, though some new potential techniques to achieve this have recently emerged [6,7]. In addition, BNNT is bio-compatible, as it has been found to have no adverse effects on living cells [8]. It also offers a higher resistance to oxidation [9,10] and a higher thermal stability [11] in comparison to CNTs. On the contrary, irrespective of their chirality, BNNTs are found to have large electronic band gaps (∼3–5 eV) [1,2]. This observed large band gap and the lack of experimental control in synthesizing BNNTs initially dissuaded researchers from working in this field. However, the development of viable synthesis techniques [12–20], together with various band gap modulating methods [21–29] have rekindled the hope in recent years and brought BNNTs into the forefront of material science research. Currently, several approaches, such as electric fields as an external stimulus [21,22] functionalization [24,25] doping [26,27], and filling [28,29], have been used extensively for tuning the band gap of BNNTs. For example, using a transverse electric field of 0.19 V/Å, the band gap of a (22, 22) BNNT is shown to vanish completely [21]; functionalization of BNNTs with fluorine atoms is found to increase their conductivity by three orders in magnitude [25,30]. In addition to the band gap modulation, functionalization of BNNTs with different molecules have been shown to disentangle and unbundle the multi-walled BNNTs [31–34], as observed in CNTs [35–38] and graphene [39]; this is an important first step toward their practical application.

Furthermore, functionalization and doping have been shown to induce *d*-electron free (*sp*-electron) magnetism in BNNTs [40–44], which offers new opportunities for their usage in spin-based electronics (spintronics). The advantage of using a *d*-electron free magnetic entity is that it would exhibit magnetism at higher temperature [45,46], which is a highly desirable property for application in spintronics. Spintronics, which requires controlled transport of spin polarized carriers, has been explored in low dimensional materials such as organic molecules [47–53], graphene [54,55], and CNTs [56–59]. However, spintronics in the BNNT is relatively a new concept. Though the effect of the hyperfine interaction [47] arising from the interaction of nuclear and electronic spins cannot be ignored in spin dephasing, the weak spin-orbit interaction in BNNT provides an important advantage for its application in spintronics; the spin-orbit interactions [47] scale as Z^4^, where Z is the atomic number, so low Z materials like B and N have weaker spin-orbit interactions. There are several excellent reviews available on the synthesis, characterizations, and applications of BNNTs [5,60–67]. In this short review article, our focus is to explore the possible applications of BNNT in spintronics.

The rest of this article is organized as follows: in Section 2, for completeness, we briefly review the electronic structure and the techniques used to tune the electronic structure of BNNTs. In Section 3, we describe the magnetic and magneto-transport properties in BNNTs. Finally, we conclude this review with a brief outlook.

## 2. Electronic Structure

**Figure 2.**
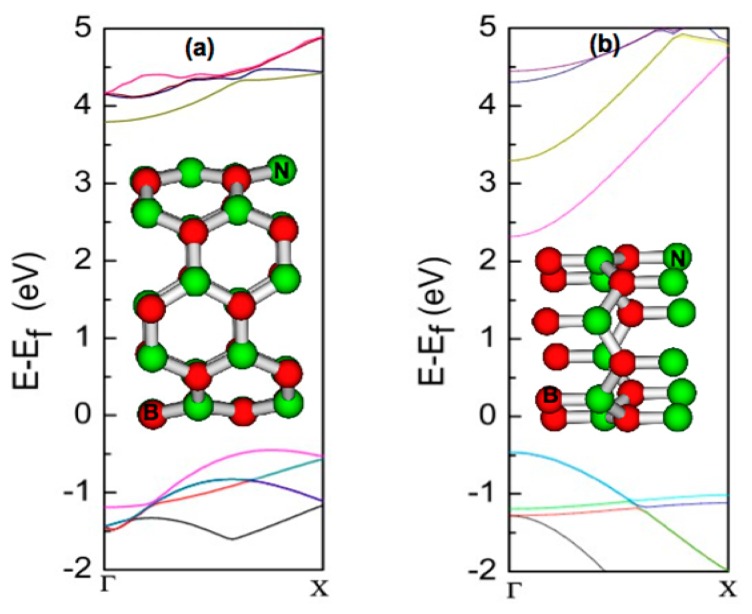
Electronic band structure of (**a**) armchair (5, 5) and (**b**) zigzag (6, 0) BNNTs; unit cell structures for (5, 5) and (6, 0) BNNTs are shown in the insets of Figure 2a and 2b, respectively. In the zigzag case, the minimum energy point in the conduction band and the maximum energy point in the valence band appear at the same Γ-point (direct band gap). In contrast, for the armchair tube, the valence band maximum shifts away from the Γ point leading to an indirect band gap. Band structures are calculated by using density functional theory; the generalized gradient approximation (GGA) with the PW91 functional for the exchange-correlation is used. The projected augmented wave (PAW) approach is used to describe the valence-core interaction.

Unlike the C-C bonds in CNTs, the B-N bonds in BNNTs are the mixture of ionic and covalent bonds [64]. Because of the ionic nature of B-N bonds, BNNTs are large band gap semi-conductors with an indirect gap for armchair and a direct gap for zigzag BNNTs [2,64]. The band structures for typical armchair and zigzag BNNTs are shown in Figure 2. The lowest conduction band is found to be parabolic in both cases and thus behaves as free electron-like [2]. The magnitude of the band gap in BNNTs is strongly affected by the nature of B-N hybridization [1,2]; the curvature induced hybridization change leads to smaller band gap with the decrease in diameter. When the tube diameter increases beyond 10.0 Å, the curvature effect diminishes and the band gap remains unchanged with diameter [1,2]. The large band gap of the BNNT limits in some instances its direct application in electronics. The range of applications of BNNTs would substantially increase if the band gap can be tuned to a desirable value in a controlled manner. Several methods for tuning the band gap of BNNTs have been successfully demonstrated [21–29]. In the following section, we briefly discuss these techniques.

### 2.1. Electric Field as an External Stimulus

One of the possible ways of tuning the band gap of BNNTs is by using a transverse electric field, which breaks the symmetry of the electronic states in the direction of applied field and mixes the nearby sub-bands in the conduction band complex and the valance band complex separately [21]. As a result, the bottom of the conduction band moves down and top of the valance band moves up, causing a reduction in the band gap of BNNTs [21]. This effect is diameter dependent; a bigger diameter BNNT exhibits a stronger response to the transverse electric field in comparison to a smaller diameter tube [21]. For example, the band gap of a BNNT with diameter 49.9 Å vanishes for a transverse field of 0.1 V/Å. In the case of a 22.2 Å diameter, a higher transverse field of ∼0.3 V/Å is required to completely close the gap [21]. Guided by the above theoretical predictions, Ishigami *et al.* first demonstrated the Giant Stark effect in BNNTs experimentally [22]. They used the tip of a scanning tunneling microscope to apply transverse electric fields and study the influence of these electric fields on the electronic properties of BNNTs [22]. Band gap modulation in semiconducting CNTs by applying transverse electric fields has also been observed [68–71]. A comparative study of the electronic structure of CNTs and BNNTs under the effect of applied transverse electric field shows a monotonic decrease in band gap with the increase of transverse electric field in the case of BNNTs [68]. In contrast, a non-monotonic change in band gap with transverse electric field (**ε_g_**) is noted in CNTs; almost no change in band gap is observed up to a certain critical **ε_g_**, beyond which a sudden transition to metallic state is reported [68]. The different responses of BNNTs and semiconducting CNTs to the transverse electric field are attributed to their different bonding features. The ionic nature of the B-N bonds in BNNT is responsible for the monotonic change in band gap in BNNTs with applied **ε_g_** [68]. In contrast, a critical **ε_g_** is required to induce polarization in C-C bonds in semiconducting CNT beyond which a stronger response to **ε_g_** is imminent. Due to the semiconducting nature of BNNT, it is also expected to be a suitable candidate for optoelectronic applications [72–77]; tuning of optical properties of BNNTs upon applying the transverse electric field has also been reported [78–80].

### 2.2. Radial Deformation

**Figure 3.**
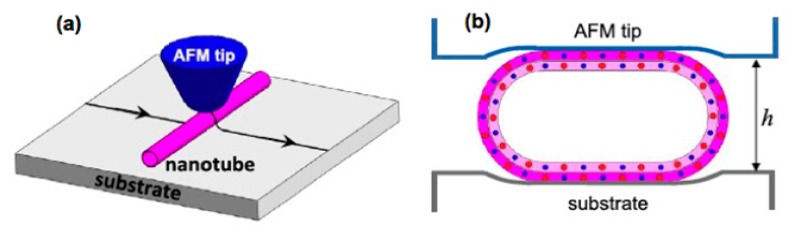
(**a**) Schematic of the AFM-based compression scheme for the radial deformation of individual BNNT on a flat substrate. (**b**) Schematic diagram of a radially deformed BNNT between an AFM tip and a substrate. Reprinted with permission from [87]; © 2012 IOP PUBLISHING.

Modulating the electronic structure of a material by applying an external pressure/strain is a common technique which has been used successfully in the past few years in different nanomaterials [81–84]. The effect of external strain depends upon the elastic properties of the materials. BNNTs and CNTs are rigid along the tube axis, but they are highly flexible along the perpendicular direction of the tube axis and have very high Young's modulus values [23,85,86]. As a result, they can sustain remarkable deformation along the perpendicular direction. Significant modification in their electronic structure is expected under a strong deformation within the elastic limit. Experimentally, radial deformation can be employed in NTs by pressing them between the AFM tip and the substrate, as shown in the Figure 3 [87]. Very recently, Ghassemi *et al.* have studied the effect of radial strain on BNNTs experimentally and have observed that the resistance of the tube decreases from 2000 to 769 MΩ and carrier concentration in BNNTs increases from 0.35 × 10^17^ to 1.1 × 10^17^ cm^−1^ upon application of a 2.5% strain [88]. On the theoretical front, several groups [23,89–91] have studied the effect of strain on the electronic structure of BNNTs. For example, using a first-principles approach, Kim and colleagues have studied the electronic structure modulation for both zigzag (9, 0) and armchair (5, 5) BNNTs under the radial deformation [23]. It has been shown that in a zigzag BNNT, the radial deformation that causes a pressure of ∼10 GPa decreases the band gap of the tube from 5 eV to 2 eV; however, in an armchair tube, the same pressure is found to have only a very nominal effect on the band gap [23]. A similar effect of strain on band gap in BNNTs has also been observed by other groups [89–91]. It should be noted that band gap modulation and conductivity enhancement upon application of radial strain are being reported in semiconducting CNTs [92–95]. The piezoelectric effect, which is a process of generating electrical charge using mechanical force, has also been demonstrated both theoretically [96–99] and experimentally [100] in BNNTs for possible applications in nanomechanical sensors and actuators.

### 2.3. Functionalization

**Figure 4.**
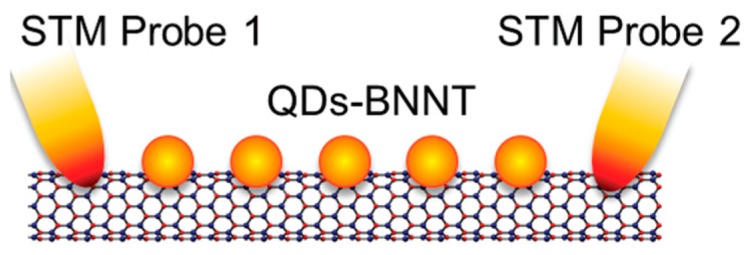
A Schematic diagram of a gold nanoparticle-decorated BNNT based room temperature tunneling field effect transistor. Reprinted with permission from [107]; © 2013 WILEY-VCH Verlag GmbH & Co. KGaA.

An alternative approach for tuning the electronic property of BNNTs is surface functionalization with different atoms, molecules, and nanoparticles [61,64]. Surface functionalization not only modifies the band gap of BNNTs, but also makes them soluble in several solvents [24]. Functionalization even reduces the work function of BNNTs significantly, which facilitates the field electron emission from the tube surface [101]. Nanoparticle-decorated BNNTs [102–107] have been explored for conductance enhancement, modification of field emission behavior [103] and designing room temperature tunneling field effect transistors (Figure 4) [107]. Due to these advantages, functionalization of BNNTs has been the subject of intense research over the past few years [5,61,64]. Functionalization of BNNTs can be done in two ways, namely covalent (chemisorption) and non-covalent (physisorption) functionalization [61]. Here, we will discuss them briefly.

#### 2.3.1. Covalent Functionalization

**Figure 5.**
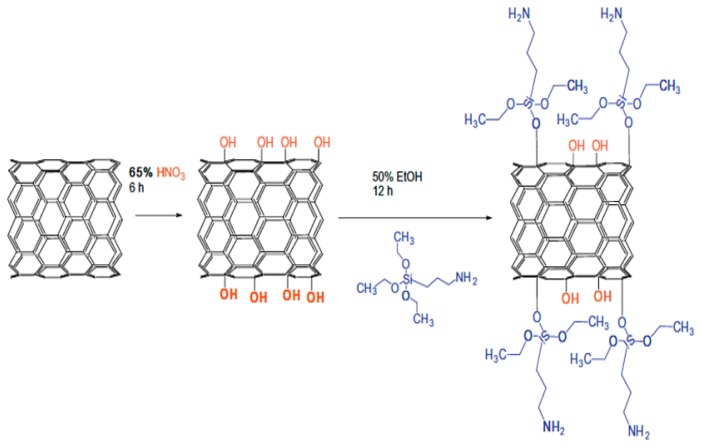
A schematic representation of the chemical functionalization of BNNT surface with 3-aminopropyltriethoxysilane (APTES) molecules. Reprinted with permission from [115]; © 2012 Elsevier Inc.

When an adsorbate affects strongly the hybridization at the adsorption sites in a host, the functionalization is referred to as covalent functionalization or chemisorption [32,61]; electronic structure modification is relatively stronger in such covalent functionalization. For example, the chemisorption of F on BNNT surface changes the *sp*^2^ hybridization at the adsorption site to *sp*^3^ [108–110]. A strong charge transformation happens in between adsorbates and BNNTs during the chemisorption, which makes covalently functionalized BNNTs either a p-type or an n-type semiconductor, depending upon the electronegativity of the adsorbates [109,111]. There are numerous schemes being published for the functionalization of CNTs [35–38] and graphene [39]. However, for BNNTs and h-BN sheets, little has been reported [60,61]. Due to the very low inherent chemical reactivity of the BNNT surface, several techniques that are used for the functionalization of CNTs and graphene are found to be unsuitable for the BNNTs [60,61,112,113]. Despite the challenges, several groups have successfully demonstrated functionalization of BNNTs in recent years [24,111–116]. For example, Bando and colleagues have demonstrated covalent functionalization of BNNTs with long alkyl chains. Comparison of the UV/Vis absorption spectra of a pristine BNNT and a BNNT with alkyl chains shows a shift of the main absorption peak of the BNNT toward a smaller value upon chemisorption as in a doped BNNT [24]. The same research group has also functionalized BNNTs covalently with different molecular groups, namely amino and -COCl groups [111]. They have observed a similar shift in the adsorption peak towards the smaller value upon the covalent functionalization as observed for the alkyl chains [24]; the theoretical study confirms that the shift in adsorption peak is due to the presence of new eigenstates around the Fermi energy arising from the adsorbed molecules [111]. Later, using ammonia plasma irradiation, BNNT surface functionalization with amine groups has been reported [112]. It has been shown theoretically that the chemical functionalization with amine groups reduces the band gap [117–119] and makes the functionalized BNNT a p-type semiconductor [117]. Recently, using a simple scheme that involves strong oxidation of BNNT with nitric acid, followed by silanization of the surface using 3-aminopropyltriethoxysilane (APTES) molecules, researchers have successfully demonstrated chemical linking of amino groups to the BNNT surface (Figure 5) [115].

A conductivity measurement of fluorinated BNNTs reveals a significantly higher conductance in the fluorine-functionalized BNNT than that for the pristine BNNT [25]. Recently, the chemical functionalization of BNNTs with different metals atoms (Sc, Ti, V, Cr, Mn, Fe, Co, Ni, Cu, Zn, Pd, and Pt) [120], and first row atoms (Li, Be, B, C, N, and F) from the periodic table [121] have also been studied using first-principles approaches. The adsorptions of these different atoms and molecules introduce impurity states within the band gap of BNNT [43,111,120–123]. In addition, like CNTs [124–126], BNNTs have also been explored as potential materials for hydrogen storage [127–133]; BN nanotubes have been demonstrated to retain 1.8–2.6 wt% hydrogen under ∼10 MPa at room temperature [128]. Electronic structure calculations show the hydrogen adsorption on BNNTs is curvature dependent [131].

#### 2.3.2. Non-Covalent Functionalization

**Figure 6.**
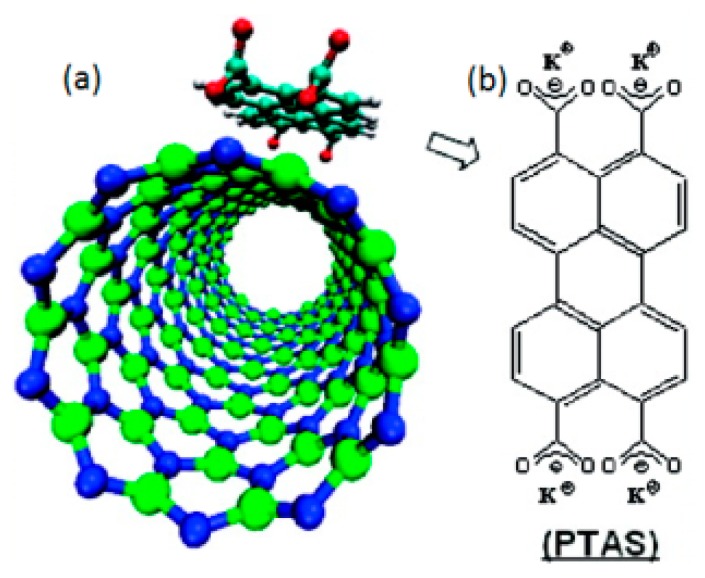
(**a**) A schematic diagram of non- covalent functionalization of BNNT with a PTAS molecule; (**b**) PTAS molecule. Reprinted with permission from [134]; © 2008 American Chemical Society.

The functionalization in which hybridization at the adsorption site of the host remains unchanged upon functionalization is called non-covalent (physisorption) functionalization [32,61]. Physisorption changes the electronic structure, while preserving the intrinsic desirable properties [32]. Unlike the covalent functionalization, non-covalent functionalization is mediated by weak interactions involving π electronic states [39]; excellent descriptions of various types of weak interaction involving π states are discussed in details in Refs. [39] and [5]. In the case of BNNTs, the π- interaction between the molecules and the BNNT surface is mostly responsible for the physisorption interaction [34,134]. For example, Wang *et al.* have reported a non-covalent functionalization of BNNTs with an anionic perylene derivative, namely PTAS, through π-stacking [134]; they observed a red shift in the adsorption spectra upon the functionalization, implying a strong interaction between the PTAS and BNNTs (Figure 6) [134].

Guided by the above experimental report, Gao *et al.* carried out a first-principles study [135] on the BN layers non-covalently functionalized with perylene derivatives and found that the weak van der Waals interaction between the adsorbate and the host BN layers mediates this functionalization. An important finding of this work [135] is that although the interaction between BN layer and PTAS is of the van der Waals type, the band structure of the physisorbed system is different from the superposition of the band structures of the host BN layer and the PTAS adsorbate. This suggests that the physisorption of BNNTs with PTAS can effectively modify the electronic structure of BNNTs around the Fermi energy [135].

**Figure 7.**
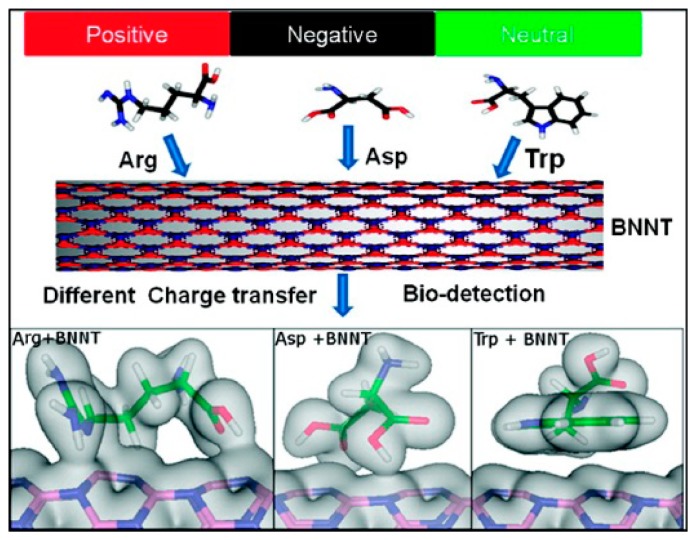
Non-covalent functionalization of BNNT with polar and non-polar amino acids. Trp (non-poar), Asp (polar), and Arg (polar) stand for tryptophane, asparatic, and argenine, respectively. The upper panel shows the charge transfer between the BNNT and amino acids, and the lower panel represents the iso-surface charge density of amino acids functionalized BNNT system. Reprinted with permission from [138]; © 2008 American Chemical Society.

Recently, different types of aromatic molecules have been used to functionalize BNNTs non-covalently [136,137] for possible applications in field effect transistors; thus, adsorption of an electrophilic (nucleophilic) molecule on BNNTs makes them p-type (n-type) semiconductors. Using density functional theory, Peyghan *et al.* have also reported that the work function of BNNTs is reduced significantly via functionalization with 1,2 diaminobenzene (DAB) [101]. Interaction between different biological molecules such as amino acids [138] (Figure 7) and nucleic acid bases [139] with BNNT surfaces have also been investigated using density functional theory; polar amino acids are found to exhibit a relatively stronger interaction with the BNNT surface in comparison to non-polar ones [138].

In the case of nucleic acid bases, guanine is found to have stronger binding with the BNNT surface than the other nucleic acid bases [139]. In addition, various other molecules, such as CO [140], H_2_ [140], isoniazid (INH) [141], metalloporphyrins [142], and 2,4,6-trinitrotoluene [143], have also been explored for non-covalent functionalization of BNNTs.

The pristine BNNTs are usually entangled and they form a bundle during experimental synthesis due to strong van der Waals interaction between the tubes; they are also rarely soluble in organic and aqueous solutions [31–34]. For practical applications, one needs to separate the individual BNNT from the bundle. This can be done using both covalent and non-covalent functionalization; however, the non-covalent one seems more appropriate since it preserves the intrinsic properties of the BNNTs [32]. Different conjugated molecules, such as poly[*m*-phenylenevinylene-*co*-(2,5-dioctoxy-*p*-phenylenevinylene)] (PmPV) [144] perylene-3,4,9,10-tetracarboxylic acid tetrapotassium salt (PTAS) [134], flavin mononucleotides (FMN) [31], peptide [34], poly(xylylene tetrahydrothiophenium chloride) (PXT) [32], poly(dodium vinyl sulfonate) [32], and poly(*p*-phenylene-ethynylene)s [145], have been used to make BNNTs soluble in different polar and non-polar solvents through functionalization. In addition to normal polymers, non-conjugated molecules such as denatured DNA [146] and microperoxidase-11 (MP-11) [147] have also been used to disperse BNNTs in aqueous solution. The interaction of glycine with BNNTs has also recently been shown to unbundle the bundled BNNTs [33]. On the theoretical front, conjugated molecules, such as poly[*m*-phenylenevinylene-*co*-(2,5-dioctyloxy-*p*-phenylenevinylene)] (PmPV), polystyrene (PS), and polythiophene (PT) [148], [*p*-(1,1,3,3-tetramethylbutyl)phenyl ether (Triton X-100) [149] are also explored for non-covalent functionalization of BNNTs.

### 2.4. Defect, Doping, and Filling

Like CNTs [150], BNNTs also possess different types of defects during experimental growth, such as vacancies, foreign atom substitutions, and Stone-Wales (SW) defects [102,151–158]. Defects modify the electronic structure and mechanical properties of the BNNTs. Experimentally, it has been shown that vacancies and extended topological defects in the BNNTs can be formed by electron irradiation [157]. SW defects are predicted to be formed upon applying a large strain [153,155,158]. Electronic structure modifications of BNNTs due to single and double (di) vacancies, and SW defects have been studied extensively using first-principles approaches [41,151–156,158]. In the case of single/di vacancies, the *sp^2^* hybridization at the vacancy site changes [154] and new eigenstates appear near the Fermi energy [41]. However, no such modification in electronic structure has been observed due to SW defects [155], which preserve the *sp^2^* hybridization at the defect site.

**Figure 8.**
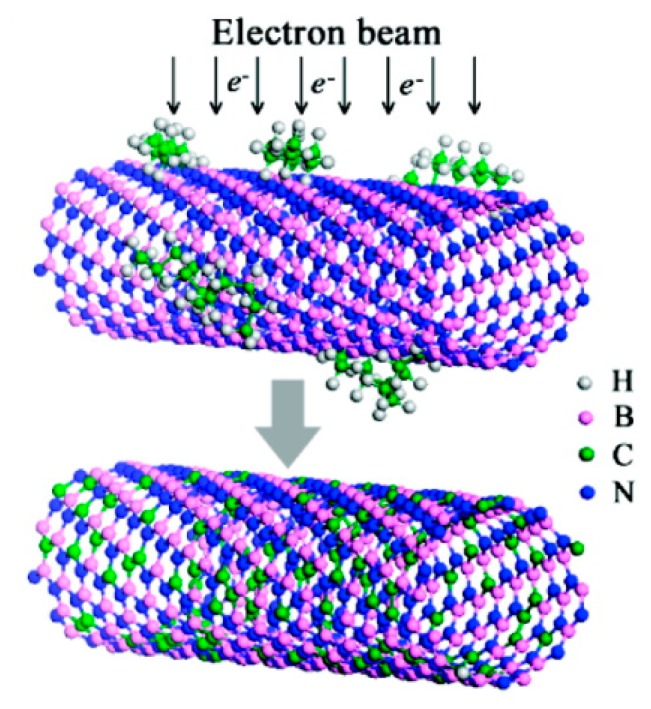
A schematic diagram of the electron-beam-induced C doping in BNNT. Reprinted with permission from [27]; © 2010 American Chemical Society.

Doping in BNNTs can be done either by introducing purposefully the foreign atoms during the growth of BNNTs [25,26,159,160] or by replacing the host atoms (B or N) by foreign atoms after the synthesis of BNNTs [27,161,162], which is known as post-synthesis doping. The former approach is very popular and has been used extensively for doping of BNNTs and CNTs [25,26,159,160]. Recently, Chen *et al.* have used this approach for doping Eu atoms in BNNTs [26]. However, the advantage of the post-synthesis doping technique is that it can place a specific dopant into a precise location in the BNNT though masking/lithography, but this technique also has some disadvantages due to the very high temperature requirement for the diffusion and the damage caused by ion implantation [27]. Very recently, Wei *et al.* have successfully used an electron-beam irradiation post-synthesis doping technique to dope C atoms on BNNTs (Figure 8) [27], which is claimed to be well-controlled, workable at room temperature, and to cause minor damage; the conductivity of C-doped BNNT is found to depend upon the concentration of C dopants, which is controlled by irradiation time [27,162]. On the theoretical front, different types of dopants, such as C [41,163–167], O [41,168–170], Si [171,172], Ge [173], and F [110], have been used to study the electronic structure of doped BNNTs. These calculations have revealed that the doping essentially introduces new eigenstates near the Fermi energy resulting in a modification of the electronic structure [25–27,159–162]. Transition metals (TMs) have also been used for doping the BNNTs; TM-atom doping not only changes the electronic structure significantly, but also increases the reactivity of the BNNTs' surface [174–177]. The large amount of charge transfer from the TM atoms to the BNNT is responsible for the significant change in the electronic structure in TM-doped BNNT [174–177].

Due to the large band gap and chemical inertness of BNNTs, they have also been explored as shielding materials for metallic nanowires (NWs) [178]; metallic NWs are metastable and need to be stabilized from the outside interference without affecting their intrinsic electronic properties for applications in nanoelectronics. Recently, different groups have successfully demonstrated encapsulation of various nanostructures such as Mo clusters, Ni, Co, Fe, Mn, β-SiC and Fe-Ni nano-wires/rods in BNNTs [29,178–186]. Theoretically, it has been shown that there is a weak interaction between the BNNTs and encapsulated nanowires/rods [187–191]; the contribution to the density of states near the Fermi energy in the hybrid system comes from the encapsulated nanowires/rods [188], which suggests that the BNNT can be used as an ideal insulator wrap around a metallic conductor. Theoretical study also reveals that the encapsulation of an electrophilic molecule in BNNT introduces new acceptor states close to the valence band edge of the BNNT making it a p-type semiconductor; encapsulation of nucleophilic molecule in BNNT does not bring about much change in the electronic structure near the Fermi energy [192,193]. BNNTs of different diameters have also been used to pack the C_60_ molecules [28]; theoretical study shows that (10, 10) and (17, 0) BNNTs are the most favorable size for the encapsulation of C_60_ [194].

## 3. Magnetism

### 3.1. Static Magnetism in BNNTs

Traditionally, the formation of local magnetic moments in different elements and alloys has been associated with partially filled *d* and *f* states of the elements [45,46,195–197]; collective magnetism in such materials is the result of coupling between these localized moments. However, the possibility of local magnetic moment due to *s* and *p* states cannot be ignored [195]. Magnetism in different metal- free (*sp*) materials has been reported recently both theoretically and experimentally [45,46]. The main advantage of the metal-free magnetic materials is that they have high Curie temperatures [46]. As a result, they can be utilized for room temperature spintronic applications. Initially, the *sp* magnetism was observed in carbon-only materials [46]. Later, this observation is extended to other materials, such as CaB_6_ [45], GaN [195], BN [195], CaB_2_C_2_ [198], g-C_4_N_3_ [199], and CaO [200]. It has been shown theoretically that electronic instabilities caused by bonding defects in various materials are responsible for the *sp* magnetism [199]. There is an excellent review article on magnetism in C-based materials [46]. Here, we briefly review *sp* magnetism in boron nitride nanotubes.

As mentioned above, local magnetic moments in BNNTs arise from atoms with unsaturated valance electrons or dangling bonds, which can be created purposefully in a controlled manner either by introducing defects or by doping/adsorption (adatom) of BNNTs with different foreign species [42,158,174]. One of the most probable defects in BNNTs is the vacancy [41,151,153], which can either be an N vacancy (V_N_) or a B vacancy (V_B_). Initially, Schmidt and colleagues reported magnetism in BNNTs with V_N_ and V_B_ using spin polarized density functional theory. They observed an exchange splitting of ∼0.5 eV for both the vacancies [151]. The comparison of formation energies between the V_N_ and V_B_ in BNNTs shows that the V_N_ state is more favorable than the V_B_ state [41]; however, both the vacancies induce spontaneous magnetization with a magnetic moment of 1 μ_B_ per each defect [41]. In the case of the V_B_ state, the unpaired electron from the unsaturated N atom contributes to magnetism. However, in the V_N_ state, unpaired electrons from both the unsaturated B atom and the neighboring N atoms in the vicinity of the defect contribute to the magnetism [153]. Di-vacancies on the other hand do not induce magnetization in BNNTs [153]. There are no reports on magnetism due to SW defects. Like BNNTs, single vacancies in CNTs are reported to induce magnetism; CNTs with di-vacancies do not exhibit magnetism due to reconstruction at the defect site [201,202].

Substitutional doping (SD), which is an another avenue of inducing magnetism, has been explored in BNNTs. SD of transition metals such as Co, Ni, Cr, Mn, Fe, Cu, V, Ti in BNNTs has been shown to exhibit strong magnetism [174,175]; however, the very short spin relaxation time of TM atoms due to their large spin coupling hinders their application in spintronics [199]. To address this challenge, SD with nonmagnetic atoms has been explored to induce magnetism in BNNTs [40,41,162–167]. For example, SD of carbon in BNNTs has been found to induce strong magnetism with enhanced conductivity [40,162–167]; the antiferromagnetic coupling between local magnetic moments has been found to be the stable configuration in C-doped BNNTs [163]. Furthermore, the magnetism in C-doped BNNTs is found to be independent of chirality and doping site; doping of the C at either the B or the N site yields a magnetic moment of 1 μ_B_ per defect [40,41]. In addition to substitutional doping of C, C-BN hetero-structured NTs have been shown to exhibit magnetism at the interface of the C and BN nanotube segments [203]. It should be noted that magnetism is also observed at the interface of graphene/h-BN heterostructures [204,205] as observed in C-BN heterostructured nanotubes [203]. Si and Ge atoms, which have the same outer shell electronic configuration as C, have also been used for substitutional doping of BNNTs to induce magnetism [172,173]; the substitution of either B or N by Si/Ge yields a magnetic moment of 1 μB in BNNT per substitution; 3*p* electrons from Si and 4*p* electrons from Ge are mainly responsible for the induced magnetism in Si- and Ge-doped BNNTs, respectively [172,173]. Unlike C doping [40,41], Si and Ge doping have been found to distort the cylindrical shape of the BNNTs [172,173]. Substitutional doping with oxygen is also reported to induce magnetism in BNNTs [170]. In all the cases, substitutional doping in BNNTs essentially introduces dispersionless energy levels near the Fermi energy, suggesting that the corresponding electronic states and magnetic moments are highly localized [40,41,162–167,172–175].

**Figure 9.**
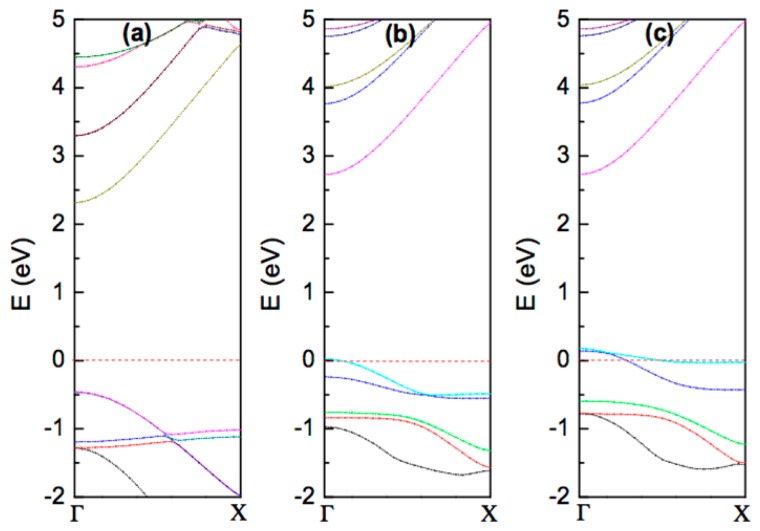
Electronic band structure of (**a**) pristine (6, 0) BNNT, (**b**) fluorinated (6, 0) BNNT (spin up states), and (**c**) fluorinated (6, 0) BNNT (spin down states); the Fermi-energy is set to zero. The partially filled bands near the Fermi-energy in the case of fluorinated BNNT have small dispersion as the fluorine atom acts as a shallow acceptor upon adsorption on BNNT. Band structures results presented here are obtained using density functional theory; the generalized gradient approximation (GGA) with the PW91 functional for the exchange-correlation is used. The projected augmented wave (PAW) approach is used to describe the valence-core interaction.

Adsorption of different atoms on the BNNT surface can lead to modification of the electronic structure of BNNTs [108–119], and can induce magnetism in BNNTs [30,42,43,120,122] as seen in CNTs [206–208], but, to date, besides TM atoms [120,122], only adsorption of three different atoms, namely C, B and F, have shown to induce magnetism in BNNTs [42,43,209,210]. In the case of C adsorption on the BNNT surface, a local magnetic moment of 2 μ_B_ is observed at the ad-atom site regardless of the diameter of the tube; two valence electrons of the adsorbed C atom make a bond with the host atoms and the two remaining unpaired electrons contribute to the magnetism [43]. From an application point of view, induced local magnetic moment alone is not enough for their usage in spintronics; ferromagnetic spin ordering (FM) between the localized moments is essential. In BNNT with C ad-atoms (C-BNNTs), the antiferromagnetic spin ordering (AFM) is found to be more stable than the FM configuration [43]. Upon the injection of extra electrons, the AFM ordering in C-BNNTs is found to switch to a FM configuration [43]. In the case of F adsorption on BNNTs, unlike the C adsorption, the F atom takes some electrons from the neighboring N atoms, and the unpaired electrons at the N sites contribute to magnetism [30]. It has been reported that the F atom prefers to adsorb on top of the B atom in BNNTs. The local deformation induced by F-adsorption changes the *sp*^2^ hybridization of a B atom in the pristine BNNT to *sp*^3^ hybridization in the fluorinated BNNT [30,42,210]. Figure 9 shows the band structure of pristine and fluorinated (6, 0) BNNTs; new energy levels for both the up and down spin states having relatively small dispersion appear close to the Fermi energy upon the adsorption of F on BNNTs. Electronic structure analysis shows that there is an asymmetry in the density of states between the spin up and spin down branches at the Fermi energy giving rise to magnetism in the F-BNNT with a net magnetic moment of 0.99 μ_B_ per F atom [30,210]. F-BNNT has also been found to exhibit long range ferromagnetic spin ordering [30,42]. The exchange coupling (**J**) between two neighboring moments, which provides the strength of the ferromagnetic ordering, has been reported to be curvature dependent; radial strain enhances the curvature effect and increases the value of J [42]. It is important to note that in the fluorinated h-BN sheet (with no curvature), the FM and AFM spin states are almost degenerate [211]. One of the advantages of the *sp* magnetic materials is their high Curie temperature, which can be estimated from the energy difference between the paramagnetic (PM) and the stable FM states [46]. Recent first-principles calculation shows that the energy difference between the PM and FM states in a (6, 0) F-BNNT with fluorine coverage of 4.1% is 1.6 eV, suggesting that the fluorinated BNNTs can be utilized for high temperature spintronics [30]. Similarly, B adsorption on BNNTs is reported to induce magnetism, which is found to be independent of the tube diameter [209]. Magnetism in BNNTs arising from the unsaturated dangling bonds at the open end of the tube has also been reported [44]; the observed magnetism is strong and is not affected much by external perturbations, such as strong electric fields and doping. This suggests that the open-ended BNNT could possibly be used as spin polarized electron field emitters [44].

### 3.2. Spin-Polarized Electron Transport in BNNTs

Thus far, we have reviewed static magnetism associated with BNNTs. However, for their applications in spin-electronics [212], one needs to understand the controlled transport of spin polarized carriers through the BNNT channel connected to semi-infinite leads. In principle, the channel (or semi-infinite lead) can be either magnetic or non-magnetic. When a channel is attached to magnetic electrodes, an applied bias drives the spin polarized electrons from the spin source to the spin drain. Because of the distinctive nature of the spin up and spin down electrons, they experience different scattering potential during the transport leading to a spin polarized current in the circuit [213]. Usually, depending upon the relative orientation of magnetization in the magnetic contact layers the circuit resistance changes from minimum resistance for the parallel magnetization (P) to maximum resistance for the anti-parallel magnetization (AP) between the contacts resulting in a spin-valve effect [45,49–53,55,212]—*The foundation behind modern high density data storage devices.* For a semiconducting channel, the relative resistance between the P and AP configurations is known as tunnel magneto resistance (TMR) [49]. The TMR value in general is higher than the magneto resistance observed in a spin-valve device with a metallic spacer, which makes the TMR device much more appealing [49] than the MR device with a metallic spacer. CNT- and graphene-based spin-valve devices have been reported both theoretically and experimentally [54–59]. Despite the large band gap in BNNTs [1,2] and the observed strong response to transverse electric fields [21,22,68], BNNTs have not been explored as spin tunnel devices until now. Using first-principles approaches, very recently, the electric field control of TMR in a BNNT junction has been predicted [213]; since the spin coherence length is expected to be longer than the channel-length considered in this study, a spin coherent conserved tunneling approach is used to determine the spin polarized current. Though thus far there is no experimental demonstration of TMR in BNNTs, it is expected that this theoretical study [213] will initiate new experimental effort towards its verification.

#### 3.2.1. BNNT as a Spin Transistor

**Figure 10.**
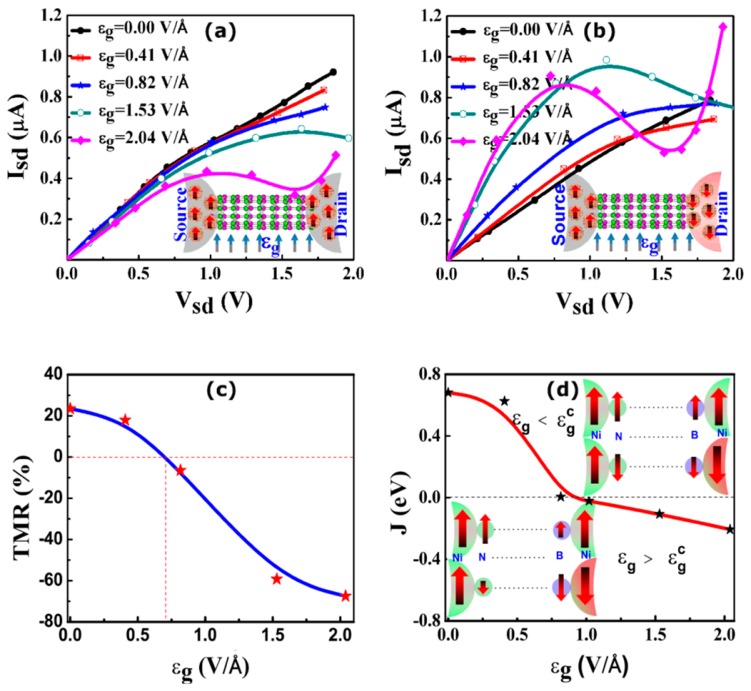
Switching in sign of tunnel magneto resistance (TMR) and exchange coupling (J) with transverse electric field (ε_g_). I_sd_-V_sd_ curves in a BNTQD tunnel junction for (**a**) parallel (P) spin configuration and (**b**) antiparallel (AP) spin configuration as a function of ε_g_. Insets show the schematic junction structures for the P and AP spin configurations. (**c**) TMR *vs.* ε_g_ at V_sd_ of 0.2 V. (**d**) Exchange coupling as a function of ε_g_. Inset shows the ε_g_ dependent spin-profiles at the BNNT/lead interfaces. The height and width of the arrow determine the magnitude of magnetic moment; higher the height/width, the more is the magnetic moment. Up and down arrows denote positive and negative magnetic moments, respectively. A singe particle Green's function approach in conjunction with density functional theory is used to calculate the spin polarized current. The TMR is calculated from the relative difference in current between the P and AP spin configuration as in the [213]. The exchange coupling is estimated from the difference in energy between the P and the AP configuration. Reprinted with permission from [213]. © 2014 the Owner Societies [213]

A spin-valve transistor is a three terminal device analogous to a traditional silicon based transistor (MOSFET); however, the source and drain in the spin-valve transistor [214] are made from magnetic materials instead of normal metals. In order to design a BNNT-based spin valve transistor, one needs to first make a BNNT spin valve by sandwiching a BNNT of finite length between two nickel electrodes. Since electrons are confined in the BNNT channel due to its finite size, and there is a lattice mismatch between the nickel electrodes and the BNNT, the channel is referred to as the BNNT quantum dot (QD) [213]. As in a typical spin-valve device, current in the parallel spin configuration (I_PC_) is reported to be higher than that in the anti-parallel configuration (I_APC_) in the BNNT spin valve (Figure 10a,b) [213]. The bias range from 0 to 2 V is considered. The TMR value, which is obtained as (I_PC_ − I_APC_)/I_APC_ × 100% [49,50,52], is found to be ∼24% at a bias of ∼0.2 V (Figure 10c), suggesting that it can be used as a two terminal spin switch. Next, the transverse electric field (ε_g_) is applied in the device to mimic the gate field in a three terminal spin transistor [213].

The schematic junction structures of BNNT-based spin valve transistors for the P and the AP spin configurations are shown in the insets of Figure 10a,b. Figure 10a,b show the variation of current (I) with bias (V) at different **ε_g_** for the P and the AP spin configurations, respectively. I_PC_ decreases and I_APC_ increases with the increase of **ε_g_** within the linear regime, resulting in a switching of TMR as shown in Figure 10c; the sign of TMR changes from positive to negative at **ε_g_** ∼ 0.71 V/Å. It should be noted that a similar gate field induced switching of magnetoresistance is observed experimentally in CNT attached to ferromagnetic electrodes [57,59]; however, the magnitude of MR in CNTs is found to be smaller than that observed in BNNTs [213]. For a stronger **ε_g_**, as expected, a non-liner feature in I-V in BNNT is observed. Molecular orbital analysis shows the frontier orbital characters (particularly at the interface) are different for the P and AP configurations. Hence they respond differently to transverse electric field [213].

Since the band gap modulation of BNNT with **ε_g_** is diameter dependent [21], as discussed in Section 2, one expects the switching of TMR to occur at a smaller **ε_g_** for a larger diameter BNNT channel. Like TMR, the exchange coupling (J), which is estimated from the energy difference between the P and the AP spin configurations, is found to switch sign from positive to negative at **ε_g_** ∼0.8 V/Å. This shows a close resemblance between the TMR and J in the BNNT spin-valve transistor [213]. The change in sign of J is attributed to the electric field-induced modification of magnetic exchange interactions at the interface [213] (see the inset of Figure 10d) caused by the Stark effect [55]. In addition, a very large spin injection factor in BNNT-Ni junction is observed [213], which suggests that the Ni/BNTQD spin-interface can act as a natural spin-selective tunnel barrier for efficient spin injection.

#### 3.2.2. BNNT as a Spin Filter

A spin filter allows all majority (or minority) spin carriers to pass through the channel while blocking the minority (or majority) spin carriers, which requires splitting of spin components. The origin of spin splitting goes back to the year 1922 when Stern and Gerlach through their ingenious experiment [215] demonstrated the quantization of spin angular momentum of the electron. They used a non-uniform magnetic field to split an atomic beam comprising Ag (5*s^1^*) atoms into two distinct beams corresponding to two different electron spin components. Currently, an alternative way of spin filtering without using external magnetic field for spin splitting has been explored [30,216–222]. In this scheme, a ferromagnetic channel is usually used between two non-magnetic electron *source* and *drain* [30,216]. An applied bias drives the electrons from source to drain via the channel, where the electrons are spin polarized and experience different scattering potentials, leading to a spin polarized current in the circuit. Currently, researchers are in search of new low dimensional materials that would exhibit ∼100% spin polarization. Different materials such as organic molecules [217–220] and insulating materials like EuS [216,221,222] as the channels have been explored to achieve high spin polarization. However, finding a material with high Curie temperature is an essential first step for any practical application in spintronics [46]. In this regard, the discovery of ferromagnetism in metal-free magnetic materials provides an important opportunity for designing a spin filter [30]. For example, fluorinated BNNT has recently been shown to have long range ferromagnetic ordering [30,42], which is found to be stable at a much higher temperature [30]. Besides, F-BNNT has been shown to have high conductivity [25], which makes it an ideal candidate for a spin filter. Here, we discuss on spin-filtering phenomenon observed recently in F-BNNT [30].

**Figure 11.**
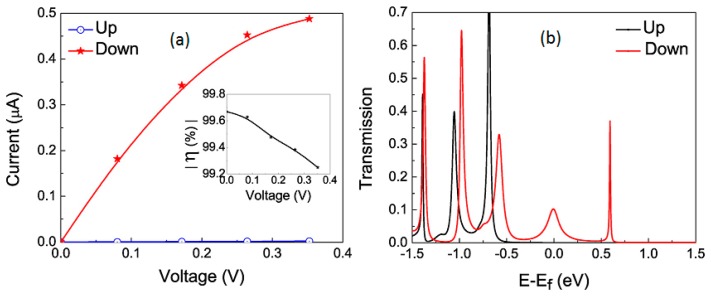
(**a**) Spin dependent current in the F-BNNT/Au junction; the F-coverage is 4.1%. Spin down current is significantly higher than the spin up current. Inset shows the variation of the magnitude of spin injection coefficient (η) with bias; the spin injection coefficient is calculated from the spin up and spin down currents as discussed in the text. (**b**) Spin polarized transmission in the F-BNNT/Au junction; the F-coverage is 4.1%; Up and Down refer to majority and minority spin states, respectively. A singe particle Green's function approach in conjunction with density functional theory is used to calculate the spin polarized current. Reprinted with permission from [30]; © 2014 American Chemical Society [30].

To design a spin filter, the F-BNNT with 4.1% coverage of F is used as the channel between two gold leads [30]. A single particle Green's function approach as developed in [223] is used to model the device. The calculated conductance in the F-BNNT junction is found to be 3.6 μS, which is about three orders higher in magnitude than that observed in the pristine BNNT-Au junction; the calculated I-V characteristics are presented in Figure 11. This result is in excellent agreement with the experimental data [25]. It should be noted that a similar low coverage of F on BNNT has been used in the experimental measurement [25,224]. Since the F-BNNT is ferromagnetic, a spin conserved tunneling approach is adopted, where the up and down spin currents are added to find the total current [30]. From Figure 11a, I_↓_ is found to be appreciably larger than I_↑_. There are two factors that contribute to the higher I_↓_ than the I_↑_. First, the location of the spin-down sub-band in the device is closer to the Fermi energy than the spin-up sub-band. The second point is that the spin-down orbital coupled more strongly to the metal state at the interface than the spin-up orbital; this increases the broadening and the escape rate of the spin-down electrons as seen from Figure 11b [30].

To quantify the difference between I_↑_ and I_↓_ in the F-BNNT junction, the spin injection factor (η) (inset of Figure 11a) is calculated as (I_↑_ − I_↓_)/(I_↑_ + I_↓_) [30]. The η value at zero bias, which is known as the spin filter efficiency, is obtained as (T_↑_(E_f_) − T_↓_(E_f_))/(T_↑_(E_f_) + T_↓_(E_f_)) × 100% [30]; T_↑_(E_f_) and T_↓_(E_f_) are the transmission coefficients at the Fermi-energy for the spin-up and the spin-down states (Figure 11b), respectively. The spin filter efficiency is reported to be 99.8% in the F-BNNT/Au junction. For a higher coverage of F (8.2%), spin filter efficiency is found to be 99.1%. Upon increasing the channel length, the spin filter efficiency increases to 99.9%. This clearly suggests that the observed high spin filter efficiency is a general feature of F-BNNT for a reasonably low coverage of fluorine [30]. Considering the experimental progress in recent years, it is expected that the spin filter proposal based on F-BNNT discussed above can be verified.

## 4. Conclusion and Outlook

Since its inception, BNNTs, which are inorganic analog to CNTs, have not received their due attention from the research community. The large electronic band gap and the challenges involved in synthesizing BNNTs have been the sole cause for its slow progress. However, aided by both theoretical and experimental progress, the understanding of the synthesis, purification, fabrication, and functionalization of BNNTs for tuning their electronic properties has advanced significantly over the past few years. For example, BNNTs have been shown to exhibit giant Stark effects. BNNTs are also being explored for possible application in nanomechanical sensors and actuators. Recently, the chemisorption of fluorine on BNNT, which enhances the conductivity of BNNT by three orders of magnitude, was found to induce long range ferromagnetic spin ordering at a temperature above the room temperature—*Opening up a new possibility of using BNNTs in spintronics*. In this short review article, we discuss the most recent advances involving BNNTs in spintronics. Both the static magnetism and magneto-transport properties in BNNT junctions are highlighted. Particularly, the dopant- and chemisorption-induced magnetism, the observation of high tunnel magneto-resistance in BNNT/nickel junctions, the demonstration of gate field-induced switching of tunnel magnetoresistance in a BNNT spin-valve transistor, and the usage of fluorinated BNNT as an ideal spin filter offer new potential routes for application of BNNT in spintronics.
